# Nocardia asteroides occipital abscess as a cause of lupus nephritis?

**DOI:** 10.1002/ccr3.5265

**Published:** 2022-02-01

**Authors:** Francis Essien, Graey Wolfley, Joshua Tate, Wayne Latack, Matthew Carroll

**Affiliations:** ^1^ Department of Internal Medicine David Grant Medical Center Travis AFB California USA; ^2^ Department of Internal Medicine Keesler Medical Center Keesler AFB Mississippi USA; ^3^ Division of Endocrinology Department of Internal Medicine Keesler Medical Center Keesler AFB Mississippi USA; ^4^ Division of Nephrology Department of Internal Medicine Keesler Medical Center Keesler AFB Mississippi USA; ^5^ Department of Rheumatology Singing River Health System Ocean Springs Mississippi USA

**Keywords:** immunocompromised, lupus nephritis, Nocardia, systemic lupus erythematosus

## Abstract

Systemic lupus erythematosus (SLE) is a multisystem disease with a complex etiology, which manifests in a multitude of manners. We present a case of lupus nephritis in a patient who developed complications of immunosuppressive treatment with eventual resolution of her nephritis following cure of her Nocardia brain abscess.

## Graphical abstract

Lupus nephritis occurs in 50% of patients with systemic lupus erythematosus (SLE) within the first year of diagnosis. Emerging research has proposed the role of infections in SLE development. We present a unique case of Nocardia and lupus nephritis.

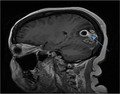

## 1 BACKGROUND/OBJECTIVE

Lupus nephritis occurs in 50% of patients with systemic lupus erythematosus (SLE) within the first year of diagnosis.^1^ Multiple theories exist with regards to the pathophysiology of lupus nephritis to include immune complex deposition, autoantibody: self‐antigen binding, and cross‐reactivity of antibodies with renal parenchyma.^2^ Prior studies have demonstrated the role of infective agents in the development of autoimmune disease. In concert with the gut microbiome, the skin microbiome is characterized by the presence of several micro‐organisms influencing the innate and adaptive immune systems. We present a unique case of lupus nephritis in a 47‐year‐old African American woman who developed complications of immunosuppressive treatment with eventual resolution of her lupus nephritis following cure of her Nocardia brain abscess.

## 2 CASE REPORT

**FIGURE 1:**
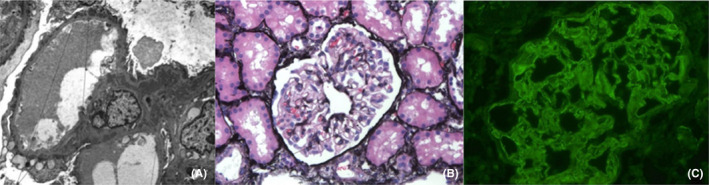
(A) Subepithelial, subendothelial and mesangial deposits; (B) Mild capillary loop prominence and subtle basement membrane irregularity on silver stain; (C) Granular capillary loop and mesangial positivity for IgG

A 47‐year‐old woman with no known history of SLE or renal disease presented with edema, proteinuria, and hypoalbuminemia consistent with nephrotic syndrome on initial presentation. Serum serology was positive for anti‐dsDNA >300 IU/ml (normal range (NR) 0–4 IU/ml), anti‐SS‐A, anti‐RNP, and anti‐SM antibodies. Laboratory data also revealed hypocomplementemia C3 74 mg/dl (normal range (NR) 82–167 mg/dl), C4 4 mg/dl (normal range (NR)14–144 mg/dl), low Ch50 <10 mg/dl (normal range (NR) 31–60 mg/dl), leukopenia, and anemia (See Tables 1 and 2 for complete biochemical evaluation). A renal biopsy was performed, which revealed findings consistent with membranous lupus nephritis (Figure 1). Histopathology demonstrated “full house” staining on immunofluorescence and deposits in all compartments on electron microscopy. The biopsy results, coupled with the patient meeting EULAR criteria for SLE, met the diagnostic criteria for Class V Lupus Nephritis. Mycophenolate mofetil 1000 mg twice daily and prednisone 60 mg daily were initiated 1 month later.

**TABLE 1:** Laboratory data on presentation 2008

WBC	1.5 (L) × 10(3)/μl	4–11
Hemoglobin	8.9 (L) g/dl	11.5–15.0
Hematocrit	24.8 (L)%	34–46
MCV	83.6 fl	80–100
Platelets	242 × 10(3)/μl	150–450
C‐Reactive Protein	39.9 mg/dl	<3
Complement C3	74 (L)	90–180
Complement C4	4 (L)	16–47
Complement CH50	<10 (L)	31–66

**TABLE 2:** Autoimmune laboratory results on presentation in 2008

Nuclear Ab panel		
DNA double strand Ab	>300 (H) IU/ml	<0–4
Smith extractable nuclear Ab	Positive (H)	Negative
Ribonucleoprotein extractable nuclear Ab	Positive (H)	Negative
SS‐A Ab	Positive (H)	Negative
SS‐B Ab	Negative	Negative
Centromere Ab	Negative <i>	Negative
Nuclear Ab	Positive (H) <i>	Negative
Urine protein	882 mg/dl	
Urine creatinine	87.19 mg/dl	

**FIGURE 2:**
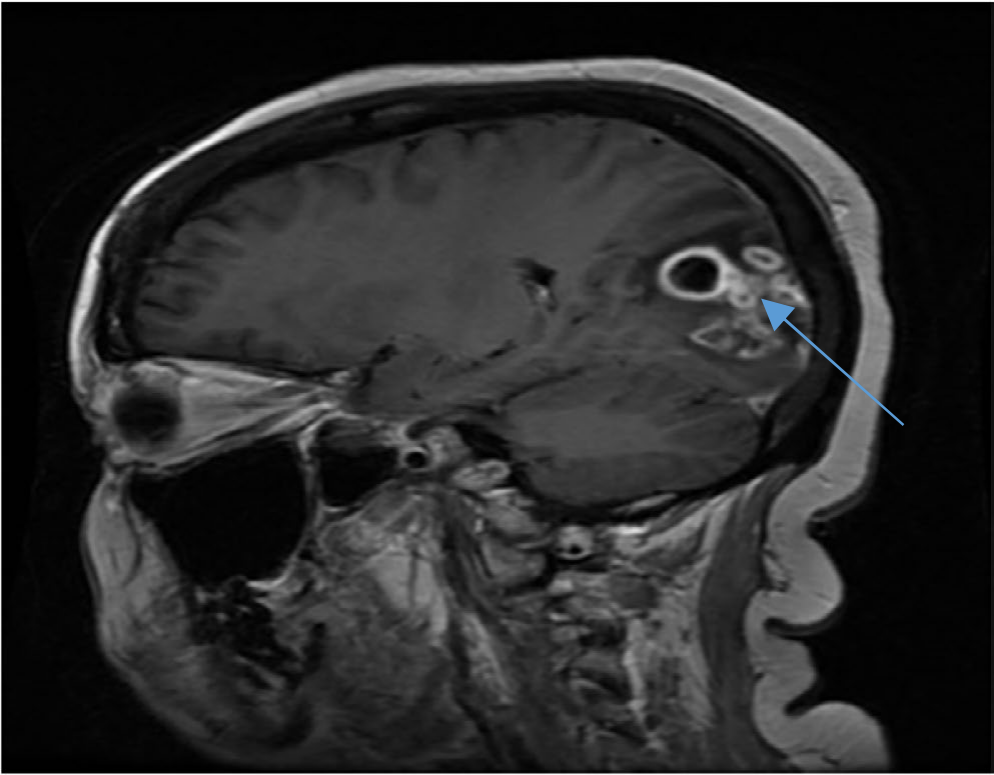
Cranial sagittal T1‐weighted magnetic resonance image showing an abscess formation in the right occipital region (blue arrow)

Patient presented for follow‐up 1 month after initiation of therapy to her nephrologist with homonymous hemianopsia, headache, nausea, and vomiting. Renal function and serology were unchanged, and proteinuria was stable at 7.5 grams/24 h. Expedited MRI brain revealed abscesses in her right occipital lobe (Figure 2). Immunosuppression was immediately stopped and she was transferred to an outside facility for neurosurgical drainage. CSF cultures demonstrated growth of Nocardia asteroides. She was initiated on intravenous meropenem and oral linezolid for an interval of 3 months with transition to oral minocycline for a total antibiotic duration of 1 year. Her occipital lobe abscesses were resolved on follow‐up with her neurosurgeon 6 months later.

**FIGURE 3:**
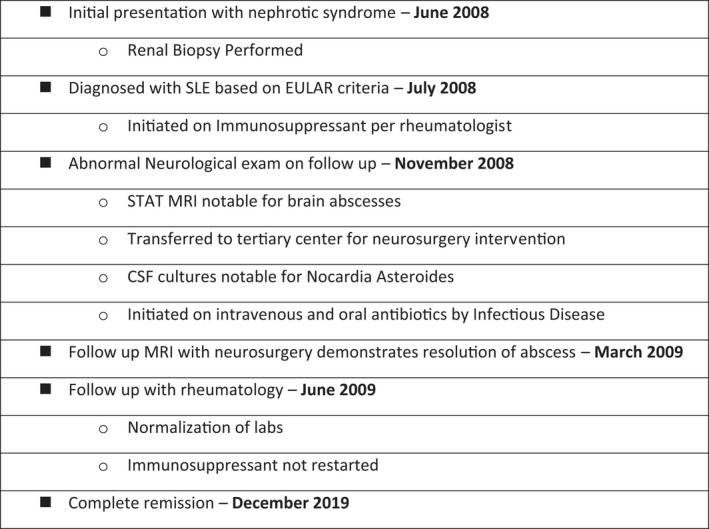
Timeline of events

Given the association of the intracranial abscess and immunosuppressed state, the mycophenolate mofetil and prednisone were not restarted. As the infection resolved, so did her proteinuria and positive serology studies except for anti‐SS‐A. On follow‐up with her rheumatologist, complement levels, nephrotic range proteinuria, anemia, and leukopenia had normalized (Table 3). A decade later after her initial presentation, the patient remained in complete remission with normal serology (Figure 3 complete timeline of events).

**TABLE 3:** Laboratory data following resolution of infection

Nuclear Ab panel		
Smith extractable nuclear Ab	Negative	Negative
Ribonucleoprotein extractable nuclear Ab	Negative	Negative
Centromere Ab	Negative	Negative
SS‐A Ab	Negative	Negative
SS‐B Ab	Positive	
DNA double strand Ab	4 <i> IU/ml	
Complement C3	166 mg/dl	82–167 mg/dl
Complement C4	23 mg/dl	14–144 mg/dl

## 3 DISCUSSION

Systemic lupus erythematosus is a multisystem disease with a natural history ranging from slowly insidious to acutely rapid and fatal.^1^ It is characterized by immune dysregulation resulting in an overproduction of autoantibodies and immune complexes. The chronic systemic inflammation affects every organ system with a highly variable clinical course.^2^ Due to increased awareness, the survival rates have improved drastically but long term prognosis is still dire.^2^, ^3^ Prevalence rates vary between 3.2 and 250 per 100,000 population, and is more commonly observed in minority populations such as Asians, African Americans and Native Americans.^3^ Females are more commonly affected with a ratio of 4:1 after puberty and there is a significantly higher rate of mortality in Juvenile SLE than adult SLE. The etiopathogenesis of SLE remains obscure but it is postulated that epigenetics plays a crucial role in the manifestation of SLE.^2^, ^3^ Epidemiologic studies demonstrate the strongest association with cigarette smoking, crystalline silica exposure, oral contraceptives, and postmenopausal hormone replacement therapy.^2^, ^4^ In certain genetically susceptible individuals, an environmental trigger will result in the loss of tolerance of self and development of immune complexes toward native proteins.

Patients with SLE are at risk of opportunistic infections like nocardiosis due to their immunosuppressed status from the disease and treatment.^5^ Nocardia species are filamentous gram positive bacteria that cause opportunistic infections with the lungs being the most common site of infection followed by skin, soft tissue, brain, joints and bone.^6^, ^7^ While Nocardial infections infrequently occur in immunocompetent patients, they primarily develop in the immunocompromised. Corticosteroids and other immunosuppressive agents are significant risk factors due to effects on cell‐mediated immunity as well as increasing risk for atypical and disseminated infections.^6^, ^8^ Nocardial infections remain uncommon; however, their incidence is increasing due to increased use of immunosuppressive medications and improved diagnostic methods. The medical literature supports this increased prevalence of nocardiosis in conjunction with SLE.^9^ Opportunistic infections remain a significant cause of death in patients with SLE and a high level of suspicion should be kept, as delay in recognition and treatment of nocardiosis increases mortality.^10^

Diagnosis of nocardiosis in patients with SLE can be complicated by Nocardial infections presenting in manner mimicking SLE or an SLE flare as demonstrated in several case reports. In one such report, Chung et al. presented a case of cutaneous nocardiosis in an immunocompetent woman with 5‐year history of a skin rash on her face and neck that was initially attributed to SLE.^11^ Another by Cheng et al reported a flare of SLE attributable to disseminated nocardiosis in a patient who was already diagnosed with SLE.^12^, ^13^ The common theme in these case reports and others is that the initial diagnoses were attributed to SLE or SLE flares until patient's did not respond to therapy and an alternative diagnosis was pursued. These cases as well as our case presented demonstrate the importance of considering Nocardia and other opportunistic infections in patients with SLE. Warnatz et al. reported a case series of patients with known systemic autoimmune disorders with CNS infections mimicking cerebral involvement of their primary diseases but on further workup seen to be of infectious etiology.^14^ A reported patient in this case series with Wegener's Granulomatosis developed CNS Nocardia with remission for 1 year off immunosuppressant therapy following remediation of the infection.

This case highlights several important aspects that are relevant to the care of all patients with SLE and the use of immunosuppressive medications. First, SLE is a complex and multisystem disease that sometimes manifests primarily targeting a single organ. A broad differential diagnosis is always prudent. Second, the treatment of SLE can present with complications, which can make management of the underlying immune disease challenging. Third, it is prudent to consider additional etiologies, which may play a role in the management of the disease. In our case, thought immunosuppressive therapy was appropriately initiated at the time of the patient's diagnosis of lupus nephritis, complete remission was only obtained through treatment of the underlying Nocardia asteroides infection.

## CONFLICT OF INTEREST

The authors report no conflict of interests.

## AUTHOR CONTRIBUTIONS

The authors confirm contribution to the paper as follows: Study conception and design: Francis Essien D.O., Matthew Carroll M.D. Data collection: Francis Essien D.O., Graey Wolfley M.D., Wayne Latack M.D. Analysis and interpretation of results: Francis Essien D.O., Joshua Tate M.D., Matthew Carroll M.D. Draft manuscript preparation: Francis Essien D.O., Graey Wolfley M.D. All authors discussed the results and contributed to the final manuscript.

## CONSENT

Written Informed consent was obtained from the patient for this study, to include use of images and is available on request.

## Data Availability

Data sharing is not applicable to this article as no new data were created or analyzed in this study.
